# Reply to Abedon, S.T. Dual-Receptor Recognition, Lysis Inhibition, Endolysin Release, and Reaction–Diffusion as Alternative Explanations. Comment on “Rojero et al. Bypassing Evolution of Bacterial Resistance to Phages: The Example of Hyper-Aggressive Phage 0524phi7-1. *Int. J. Mol. Sci.* 2025, *26*, 2914”

**DOI:** 10.3390/ijms262311468

**Published:** 2025-11-27

**Authors:** Maria Rojero, Meagan Weaver-Rosen, Philip Serwer

**Affiliations:** Department of Biochemistry and Structural Biology, UT Health, San Antonio, TX 78229, USA; rojero@uthscsa.edu (M.R.); weaverrosen@uthscsa.edu (M.W.-R.)

## Abstract

In this manuscript, we isolate and characterize a phage that displays what we call anti-bacterial hyper-aggressive behavior. This behavior appears ideal for phage therapy of bacterial disease. It includes (1) formation of semi-turbid zones that subsequently clear, (2) formation of miniature satellite plaques, which probably constitute the foundation of the semi-turbid zones, (3) multi-day enlargement of both circular plaques and cleared semi-turbid zones, and (4) non-formation of phage-resistant host colonies. We emphasize the following key details in our response. (1) The semi-turbid zones are asymmetric and occupy an area much larger (2–10x) than the area of circular plaques formed on the same Petri plate (unlike semi-turbid plaques associated with other phenomena, such as lysis inhibition and lysogeny). (2) In the manuscript’s Figure 9d, we note that phage 0524phi7-1 destroys mature colonies of the host (unlike the behavior of other aggressive phages). (3) The asymmetry of semi-turbid zones is a point that we should have emphasized (because it implies non-diffusive, energy-requiring phage transport). (4) The input of energy for phage motion can be physical (to which we add some details for two physical effects); our mentioning of phage swimming is a hypothesis (that is, however, still viable).

## 1. Introduction

Thank you for requesting a response to the seven comments made by Abedon [1]. These comments (1) are focused on the similarity of our observations to previous observations, and (2) are significant for gaining perspective on both the novelty of phenomena in the above manuscript and their use for phage therapy of bacterial disease.

In introduction, we note that similarities can exist between phenomena that have fundamentally different mechanisms and uses. For example, in the disciplines of chemistry and physics, nuclear fission, nuclear fusion, and hydrocarbon oxidation all generate heat. However, the basic mechanisms and uses differ fundamentally for all three.

The anti-bacterial hyper-aggression of phage 0524phi7-1 is associated with some phenomena like those previously observed for other phages. However, differences are sufficient to indicate a fundamental difference in both mechanism and uses, the latter for improving phage therapy of bacterial disease. In addition, novelty exists for (1) the association of several of these phenomena with one phage and (2) the resultant potential for further improving phage therapy. Our responses to the published comments will be made under the same headings that were used in the comments. The comments will be repeated in italics.

## 2. The Bypassing of the Evolution of Host Resistance

*“While I agree that this is a relevant phage characteristic, particularly for phage therapy applications, it is important to note that similar characteristics have been previously described. Borin et al. [2] in particular characterized phages with comparable properties as ‘dual-receptor generalists.’ Additionally, numerous phages have been documented to independently recognize multiple receptors, including on individual bacterial cells [3–5]. Therefore, while notable, this characteristic may not be unique to the phage described by Rojero et al. [1].*

This comment cites experiments of two types as analogous predecessors of the 0524phi7-1 bypass of host resistance. In the first, the phages are investigator-evolved to infect hosts that had previously evolved to be resistant to the wild-type phage. However, in the case of phage 0524phi7-1, the phage bypasses host resistance (if any) so rapidly that we never see a resistant host. Again, a similarity with previous phenomena exists. However, something fundamentally different is occurring for phage 0524phi7-1. We do not yet know what this is. Parenthetically, for phage therapy, we do not need to know.

In experiments of the second type, phages were found to have very broad host ranges, which was enabled by tail fibers of multiple types. These manuscripts do not focus on bypassing host resistance and are not a predecessor of our manuscript. Nonetheless, a similar multi-fiber mechanism may be at work with phage 0524phi7-1.

## 3. The Clearing of Semi-Turbid Plaques

*“Although the specific cause of the observed plaque turbidity observed by Rojero et al. remains uncertain, one possible explanation involves the lysis inhibition phenomenon seen in some obligately lytic phages [6]. Lysis-inhibited, phage-infected bacteria undergo lysis after extended infection periods, a process termed ‘lysis-inhibition collapse” [7–10]. This delayed ‘collapse’ phenomenon could account for the clearing observed by the authors. It should be noted, however, that lysis inhibition does not necessarily indicate that a phage is hyper-aggressive, but instead that it is a phage that can be inducibly slow to lyse its host. Alternatively, the semi-turbidity might result from lawn microcolonies requiring additional time to become fully infected and then fully lyse, particularly under conditions of lysis inhibition [11]. That is a possibility that may be supported by the successful isolation of bacteria following streaking from ‘semi-turbid 18-h incubation’ as reported by the authors. That is, resulting colonies perhaps originated as uninfected bacteria from within still partially intact lawn microcolonies. Note that the two possibilities presented in this section are not mutually exclusive.*

**Reply:**

Our response includes the following Key Detail from the manuscript.

Key Detail #1. The semi-turbid zones are asymmetric and occupy an area much larger (2–10x) than the area of circular plaques formed on the same Petri plate. The mechanism of semi-turbid zone/satellite plaque formation involves an increase in (usually directionally biased) phage motion, not a decrease. This increase implies that the mechanism does not require anything that would slow plaque extension. In our growth medium (described in Section 7), lysis inhibition-negative, T4 rapid lysis (r) mutants make circular plaques more than 3x the size of circular wild type T4 plaques. Lysis inhibition, although it can increase the phage T4 yield per infected bacterium (review [2]), slows plaque enlargement in our medium, unless the lysis-inhibited region of the plaque has an area over 9x larger than the area of the clear center. Thus, the data do not support either (1) a lysis inhibition-requirement or (2) a temporary bacterial resistance-requirement to generate semi-turbid plaques, as proposed in this comment. In support, (1) the semi-turbid 0524phi7-1 zones do not always have a clear area, as do lysis-inhibited wild-type T4 plaques (examples of the latter [2]), and (2) the asymmetry is a further indication that lysis inhibition is not the major underlying phenomenon (see Key Point #4 in Section 7).

Finally, the presence of host cells in the semi-turbid region (1) is not a criterion for lysis inhibition because (2) it might be caused by host-independent blocking of phage infection, for example, by phage aggregation. As the manuscript shows, phage 0524phi7-1 undergoes extreme aggregation. Nonetheless, lysis inhibition and temporary bacterial resistance might be occurring in a non-rate-limiting way. However, Key Detail #2, immediately below, makes temporary bacterial resistance unlikely.

Key Detail #2. In Figure 9d of the above manuscript, note that phage 0524phi7-1destroys mature colonies of the host. Phage T7, mentioned by Dr. Abedon, is about as host-clearing as any phage previously studied. This is also true of its relative, T3. In the case of T3 and T7, we never observe anything resembling the 0524phi7-1 mature host colony-clearing. We are unaware of this phenomenon having ever been previously reported.

## 4. The Formation of Satellite Plaques

*“While this phenomenon is visually interesting, it has been recognized for decades that phage mutants appearing on plaque peripheries may acquire rapid-lysis-like phenotypes, contrasting with the slower lysis associated with lysis inhibition. The parental phages giving rise to these mutants are themselves mutated phages, as ‘star mutants’ [12–15], though that does not necessarily rule out the phenomenon from wild-type phages. Furthermore, lysis-inhibited plaques can be substantially larger than they appear visually [6], which, if also true for phage 0524phi7-1, could at least allow spontaneous formation of such mutants outside of a plaque’s visible boundary.*

*Critical questions that warrant investigation therefore include: (i) whether phage 0524phi7-1 exhibits lysis inhibition, as might be expected for a myovirus (which phage 0524phi7-1 is); (ii) whether satellite plaques represent mutants relative to the original plaque-founding virion; and (iii) whether the apparently unlysed lawns immediately surrounding visible plaques contain still-intact lysis-inhibited, phage-infected bacteria.*

**Reply:**

Comment #4 suggests that the satellite-generating plaques might be formed by mutant phages. We note that, from the beginning, we were concerned about the uniqueness of the phage isolated. As we mentioned in Section 2.1 of the manuscript, we originally cloned four times in succession to obtain a single phage. Post the original phage cloning, we subsequently performed mutation testing by separately sub-cloning phages from semi-turbid zones/satellite plaques and clear plaques. Sub-clones of both types produced semi-turbid zones/satellite-plaques with approximately the same frequency. In genetic terms, this plaque morphology did *not* breed true. We did not, but should have, mentioned this in the manuscript. We acknowledge that a stochastically determined plaque morphology would be a significant paradigm shift, and we are actively investigating this possibility. Finally, cloning of phages in individual satellite plaques is essentially impossible because of the high density of satellite plaques and the invisibility of some of them.

## 5. Multi-Day Plaque Enlargement

*While I do not question the authors’ observations of plaque growth over long time spans, it is important to recognize that this phenomenon is not unique to this phage. The podovirus T7, for example, classically produces very large plaques [16] that, under certain conditions, appear capable of continued enlargement over greatly extended periods 70 [17]. Alternatively, this too could be a consequence of the above-noted lysis-inhibition collapse phenomenon. This would be an alternative to virions continuing to successfully infect at the perimeter of plaques over very long time frames. Instead earlier infections that resulted in lysis inhibition may over time come to visibly lyse.*

**Reply:**

This comment indicates that multi-day plaque enlargement, as seen for phage 0524phi7-1, (1) also occurs for phage T7 (we agree based on our own observations), and (2) might be the product of lysis inhibition collapse. However, lysis inhibition does not occur for T7. Increasing the multiplicity of infection eventually causes non-propagative, spontaneous lysis, not the lysis inhibition observed [2] for T-even phages. Also, unlike what happens with phage 0524phi7-1, this plaque enlargement feature is not accompanied by bypass of host resistance. Host mutants resistant to T3 are easily obtained, as are host mutants resistant to phage T7. We generated and used one of the former mutants to discriminate these two phages during tests for in-mouse blood persistence of these phages [3]. Again, similarity-to-0524phi7-1 exists but is accompanied by at least one fundamental difference.

## 6. Alternative to Swimming Phages

*“The suggestion of virion spread outside of a plaque’s visual perimeter (Section 4) may be mostly equivalent to this statement by Rojero et al. [1]: ‘Phages migrated away from the central, clear plaque region, without lysing cells while migrating.’ That spread, however, is readily explainable by mechanisms other than ‘the swimming of phage 0524phi7-1’ suggested by Rojero et al. Instead, this migration could be due simply to virion diffusion through volumes not yet occupied by lawn microcolonies [18]. This is in addition to the first phages adsorbing microcolonies not displaying lysis inhibition and therefore speedily contributing to that ongoing but otherwise invisible virion spread. That expected initial display of rapid lysis by some of these spreading phages is due to lysis inhibition being induced by virion adsorption of already phage-infected bacteria. Such ‘secondary adsorption is unlikely given the low virion concentrations [19] expected at leading edges of virion diffusion within agar. This sooner lysis at those leading edges should result in an ongoing ‘Reaction-diffusion’ process [20] of virion spread, this despite the absence of visual clearing that is a result of subsequently infecting phages displaying lysis inhibition.”*

**Reply:**

Independently of the details, this comment indicates that non-infective phage movement during satellite plaque-formation might be by virus diffusion, i.e., thermal motion, which is in a random direction. However, semi-turbid zones are asymmetric, which means that diffusion-only transport is not possible. This is concluded from both physical theory and microbe-based confirmation of the physical theory, as discussed in Key Detail #3, below. A more graphic illustration of this point is that turbid and clear circular plaques are smaller than semi-turbid zones (in the same Petri plate), even though diffusion would also be the means of transport in the circular plaques of phage 0524phi7-1.

Key Detail #3. The asymmetry of semi-turbid zones is a point that we should have emphasized. Phage diffusion cannot generate an asymmetric plaque without input of energy (which can be either physically or biologically derived). The fundamental reason is the second law of thermodynamics. This principle has been empirically validated with bacteria; it is as certain as any scientific principle. Specifically, bacteria can be sterically directed if swimming in a random direction but not if moving (again, in a random direction) by thermal motion only [4,5]. Thus, semi-turbid zone generation includes phage movement that is dependent on the input of energy. We have not independently tested the principle involved by measurements of energy.

That, of course, does not prove that phage 0524phi7-1 swims, which is a relatively speculative hypothesis. As indicated in Key Detail #4, below, alternatives exist. However, as also indicated in Key Detail #4, phage swimming is a viable hypothesis. The original manuscript discusses the point that, although the host is motile, the data are best explained by the assumption that the host cannot swim through the plaque-supporting gel used and, therefore, cannot be the source of any phage motility.

Key Detail #4. The input of energy for phage motion is required by the fundamentals. This energy input can be physical; our mentioning of phage swimming (biological) is a hypothesis. In a liquid, a physically driven motion can be generated in two ways: (1) At the surface of a liquid, motion can be driven by non-uniformity of surface tension, with the direction from low-to-high surface tension. Motion of this type is the way that the boundaries of monolayers were detected in the early studies of molecular and atomic dimensions [6]. However, again, these surface tension effects are observed in liquids. Phage plaques are formed in a gel. (2) Motion can also be physically driven by osmotic and electrophoretic effects caused by concentration gradients. Concentration gradients of bacteria-contained molecules are presumably generated when bacteria lyse in restricted regions, such as plaques. The osmotic effects of concentration gradients are usually dominant and cause motion up a concentration gradient [7]. However, this is a direction opposite to the direction of phage motion to form satellite plaques. Non-uniformity of surface tension and concentration gradient generated effects are, nonetheless, possibly active in generating phage motion. However, phage swimming remains a reasonable hypothesis.

## 7. Concluding Remarks

*It is entirely possible that phage 0524phi7-1 does not exhibit lysis inhibition. However, given the potential relevance of that phenomenon to the observed characteristics, testing for lysis inhibition in this system would be valuable. For example, see the lysis-profile experiments of Rajnovic et al. [21] where such lysis inhibition is readily observed, as well as the above-cited studies [6,7,9,19]. This suggested testing is particularly important as lysis inhibition appears to be an underappreciated behavior in contemporary phage research, despite its historical [22] and potential ecological significance [6].*

*What nonetheless makes phage 0524phi7-1 particularly intriguing is that it is a myovirus possibly displaying this ever-enlarging plaque phenotype. That contrasts with the podovirus T7, where this large-plaque behavior has been classically described. As noted, however, that phenomenon in phage 0524phi7-1 might also represent a manifestation of the lysis-inhibition/lysis-inhibition collapse phenomenon.*

**Reply:**

In response to the comment about determining whether lysis inhibition occurs for phage 0524phi7-1, in the case of lysis-inhibition-active T-even phages, r mutants are readily observed (e.g., [8]). We have never observed any true-breeding plaque morphology change despite trying. Also, (1) lysis inhibition is caused by high multiplicity of infection, sometimes at the edge of plaques of T-even phages (e.g., [2,8]), and (2) determining multiplicity of infection is not possible with phage 0524phi7-1 because of its extreme aggregation. This aggregation is a possible reason for the non-lysis (because of non-host cell binding) of some host cells. In addition, the above considerations indicate that lysis inhibition is not necessary for the hyper-spreading behavior of semi-turbid 0524phi7-1 zones observed during in-gel plating.

**Figure 1:**
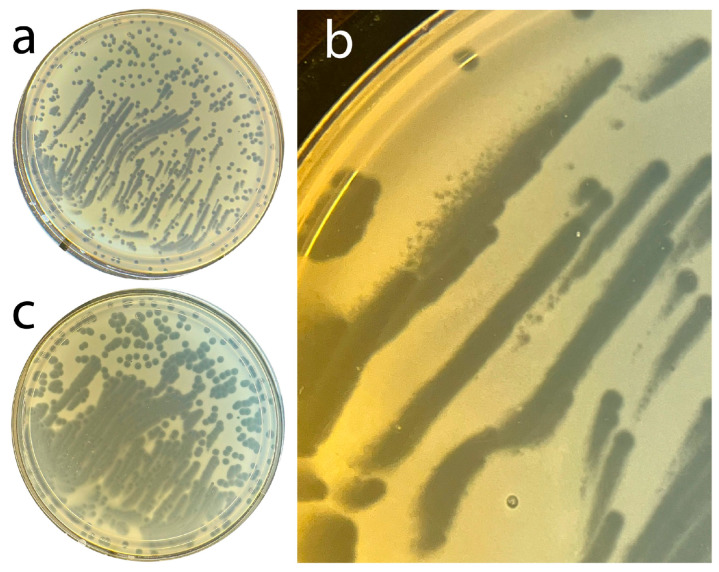
Plating of hyper-aggressive phage, T4_Ar_. Using the media described below, T4_Ar_ was subjected to plaque assay after dilution in 0.5 M NaCl, 0.01 M Tris-Cl, pH 7.4, 1.0 mg/mL gelatin, 8% polyethylene glycol 8000 (Sigma-Aldrich, St. Louis, MO, USA). The plaque assay was performed at 30 °C in a 0.4% agar (Fischer Scientific, Waltham, MA, USA), plaque-supporting gel cast above a bottom layer gel of 1.0% agar. The medium for the top layer was 4 g agar, 10 g tryptone (Fischer Scientific), 5 g NaCl in 1 L of MiliQ-water; the medium for the bottom layer was 10 g agar, 10 g tryptone, 5 g NaCl in 1 L of MiliQ-water. (**a**) Entire Petri plate, 18.0 hr. incubation, (**b**) another, magnified, partial Petri plate, 21.0 hr. incubation, (**c**) entire Petri plate in (**a**), 45.0 hr. incubation (total). The image in (**b**) was made through a magnifying glass on a plaque counter. Small white dots in (**b**) are dust particles on the glass support of the plaque counter.

What we did do in this area is to evolve phage T4 to hyper-aggression (T4_Ar_) that is similar, but not identical, to that of phage 0524phi7-1. Specifically, 5x cloned T4_Ar_, when plated from a buffer that had 6% polyethylene glycol 8000 to encourage aggregation, often formed strings of clearing, together with clear circular plaques, in a 0.4% plaque-supporting agarose gel without polyethylene glycol (Figure 1a). The strings sometimes were quasi-parallel. The string width was up to 1.5x greater than the diameter of the larger circular plaques; the string length was up to 20x greater. This all occurred in one Petri dish (Figure 1a), reminiscent of phage 0524phi7-1 at lower plaque supporting gel concentration, 0.2% (Figure 1 of Rojero et al.). The diameter of the circular plaques was larger than the diameter of even T4 r mutants. Thus, lysis inhibition was not involved. This is one criterion by which the hyper-aggression of T4_Ar_ was (1) significantly distinguished from any previously observed T4 behavior and (2) similar to the behavior of phage 0524phi7-1. Below, we describe two more such criteria. The T4_Ar_ mutant had been isolated by subjecting wild-type T4 to serial cloning, the most selective of which was cloning from colonies of phage-resistant host mutants. This selects variants capable of forming plaques on phage resistant bacteria.

Sometimes miniature satellite plaques were observed at some (not all) boundaries of strings (upper edge of the upper left string of Figure 1b). This (asymmetric) formation of satellite plaques only at some string-boundaries implied signaling, although of unknown type. Merged, asymmetrically generated satellite plaques probably formed the strings. Neither strings nor miniature satellite plaques were seen for wild-type T4.

In addition, T4_Ar_ plaques (1) continued to grow for a second day, and (2) did this without formation of resistant colonies (Figure 1c: same as Petri plate in Figure 1a). Neither (1) nor (2) occurred with either wild-type T4 or spontaneous T4 r mutants; no increase in plaque size was observed on the second day. However, resistant colonies did form after three days of incubation of the Petri plate in Figure 1a,c. A wild-type T4 plating produced almost confluent resistant colonies overnight with the same host, as seen in the Supplementary Data of Rojero et al.

As discussed above, the asymmetry of strings and their associated satellite plaques are the products of energy dependent phage motion. The *Escherichia coli* host cannot provide this motion because it is not motile. The only alternative to plaque formation associated motion is partial separation of phages during phage aggregate dissociation in pre-gelation, molten agarose solution. However, the spatial ordering to generate both strings and the quasi-parallel relationship of the strings is not expected to occur in solution where radial symmetry is dominant. Thus, this ordering is apparently caused by quasi-parallel channels in the top layer agarose, post gelation. As discussed above, this is only possible if energy-driven phage motion occurs.

We note that (1) the T4 tail has a GTPase for which function has not been assigned ([9]; review [10]). The function might be locomotion, and (2) the above results with T4 suggest that latent hyper-aggression is also present in other phages.

In summary, the hyper-aggression of phage 0524phi7-1 is novel and phage therapy-useful in that it includes all of the following: (1) spreading of lysis asymmetrically and at a speed higher than in circular plaques, (2) immediate bypass of the evolution of phage-resistant hosts, (3) multi-day expansion of zones of lysis, and (4) lysis of mature bacterial colonies. We are pursuing the fundamentals of phage hyper-aggression, while also pursuing its use for phage therapy of bacterial disease. Parenthetically, we note that the original manuscript and the above considerations imply that major adjustments are needed in both (1) environmental microbiology, given the current almost universal non-accounting for aggregating phages, and (2) phage therapy, given the current non-focus on characterizing the aggression of the phages used. We appreciate the chance to more extensively compare phenomena of hyper-aggressive phages to the phenomena reported for phages previously characterized.
